# Permissive hypotensive resuscitation in adult patients with traumatic haemorrhagic shock: a systematic review

**DOI:** 10.1007/s00068-017-0862-y

**Published:** 2017-10-27

**Authors:** Mohammed Albreiki, David Voegeli

**Affiliations:** 1 Faculty of Health Science, University of Southampton, Southampton, SO17 1BJ UK; 2 Sultan Qaboos University Hospital, Muscat, Oman

**Keywords:** Permissive hypotensive resuscitation, Hypovolemic shock, Survival, Trauma

## Abstract

**Background:**

Permissive hypotensive resuscitation (PHR) is an advancing concept aiming towards deliberative balanced resuscitation whilst treating severely injured patients, and its effectiveness on the survival rate remains unexplored. This detailed systematic review aims to critically evaluate the available literature that investigates the effects of PHR on survival rate.

**Methods:**

A systematic review design searched for comparative and non-comparative studies using EMBASE, MEDLINE, PubMed, Web-of-Science and CENTRAL. Full-text articles on adult trauma patients with low blood pressure were considered for inclusion. The risk of bias and a critical appraisal of the identified articles were performed to assess the quality of the selected studies. Included studies were sorted into comparative and non-comparative studies to ease the process of analysis. Mortality rates of PHR were calculated for both groups of studies.

**Results:**

From the 869 articles that were initially identified, ten studies were selected for review, including randomised control trials (RCTs) and cohort studies. By applying the risk of bias assessment and critique tools, the methodologies of the selected articles ranged from moderate to high quality. The mortality rates among patients resuscitated with low volume and large volume in the selected RCTs were 21.5% (123/570) and 28.6% (168/587) respectively, whilst the total mortality rate of the patients enrolled in three non-comparative studies was 9.97% (279/2797).

**Conclusions:**

The death rate amongst post-trauma patients managed with conservative resuscitation was lower than standard aggressive resuscitation, which indicates that PHR can create better survival rate among traumatised patients. Therefore, PHR is a feasible and safely practiced fluid resuscitative strategy to manage haemorrhagic shock in pre-hospital and in-hospital settings. Further trials on PHR are required to assess its effectiveness on the survival rate.

**Level of evidence:**

Systematic review, level III.

## Background

Haemorrhagic shock among severe trauma patients is responsible for early death in the pre-hospital setting and within 24 h of hospital admission [1]. Sustained hypovolemic shock after severe injury results in deleterious clinical outcomes due to impaired blood flow and oxygen delivery to tissues, including multiple organ failure, coma and possible death [2–4]. Controlling bleeding and fluid resuscitation can prevent further damage to the vital organs and minimise the potentially fatal consequences of blood loss following trauma [5, 6]. Despite the evolution of trauma science and the existence of a large number of studies conducted in the field of trauma management, fluid administration to maintain the normotensive state of post-trauma patients remains as standard care [7, 8]. Current knowledge on the optimal fluid strategy still lacks rigorous evidence from clinical trials on humans; thus huge debate exists among trauma healthcare providers about the safety of current practice regarding fluid therapy [9].

Permissive hypotensive resuscitation (PHR) is the intentional lowering of blood pressure during fluid resuscitation by restricting the volume of crystalloid fluid administered until definitive surgical control of bleeding occurs [10–12]. Restrictive resuscitation is not a new intervention in the field of trauma care, as it was proposed and tested in the 90s; however, this strategy was commonly explored in animal models and complex processes to obtain ethical approval due to the increased vulnerability of trauma subjects to a rarely explored intervention [13, 14]. The safety of PHR on a large sample of trauma patients has not been fully explored in terms of survival rate and its effectiveness in a comprehensive systematic review of the extant experiments on human clinical trials. Despite the existence of two systematic reviews, one was carried out on animal models, and the other review enrolled RCTs that focussed on exploring the required volume of bleeding trauma patients without predefined readings of initial blood pressure [15, 16].

This review intends to gather the published literature that compares the mortality rate between aggressive and hypotensive resuscitation, and to synthesise the evidence aiding the amendment of the current protocol of fluid therapy in trauma settings. The importance of conducting this review comes from being the first to retrospectively search for high-quality extant studies, which examined the associated survival rates of deliberate hypotensive resuscitation on hypotensive, traumatised subjects.

## Objective

The current review aims to comprehensively review human clinical experiments that test the survival rate among hypotensive trauma patients managed with restrictive, controlled fluid resuscitation.

## Methods

This study was conducted in a systematic review design and reported in compliance with the Preferred Reporting Items for Systematic Reviews and Meta-Analysis (PRISMA) [17].

## Research question

The research question is structured based on Population, Intervention, Comparator, and Outcomes (PICO) format. The formulated primary question is: “What is the effect of a permissive hypotensive resuscitation strategy on the survival rate of adult patients with traumatic haemorrhagic shock?”

### Study eligibility criteria

The eligibility criteria were predefined before commencing the search process of the published literature for this review. All study designs describing the mortality rate of either hypotensive or aggressive resuscitation were eligible for inclusion in this review. The inclusion criteria were as follows: (1) adult patients (aged ≥ 15 years) of blunt or penetrating trauma, with one or more documented episode of hypotension (systolic blood pressure ≤ 90 mmHg); (2) studies conducted on humans and in clinical settings, either in pre-hospital or in-hospital critical care; (3) full-text articles in English, and; (4) study outcomes that compare the survival rate between participants resuscitated with either low or large fluid administration, or that measure the number of deaths in patients managed with fluid resuscitation.

On the other hand, studies were exempted for the following reasons: (1) conducted on animals; (2) in non-clinical settings; (3) carried out in primary healthcare settings; (4) with no focus on measuring the mortality rate of participants; (5) case studies and poor methodology designs, and; (6) studies on hypotensive resuscitation in patients with traumatic brain injury (TBI), which might affect the identification of the real cause of death among participants.

### Search strategy

The eligible studies were searched using the following health-designated databases: EMBASE, MEDLINE (EBSCO), PubMed, Web-of-Science, and the Cochrane Central Register of Controlled Trials (CENTRAL). Additionally, a manual search through Google Scholar alongside examining the reference lists of eligible studies were carried out to locate additional sources of published literature. The search process was performed for a period of 3 months, from May to August 2016. The terms entered into the databases along with the number of results are illustrated in Table 1.

**Table 1:** Search strategy

Database	Query	Hits	Unique
EMBASE (OvidSP)	(Permissive hypotensive resuscitation or hypotensive resuscitation or controlled resuscitation or balanced resuscitation or low volume resuscitation or restrictive resuscitation) and (hypovolemic shock or haemorrhagic shock or hemorrhagic shock or bleeding) and (crystalloid fluid solution or isotonic fluid solution or normal saline or NS) and Trauma) * or injur* or blunt trauma or penetrat* trauma or motor vehicle collisions or road traffic accidents or wound*) not animal). ab	21,041	71
Medline (OvidSP)	((Permissive hypotensive resuscitation or hypotensive resuscitation or controlled resuscitation or balanced resuscitation or low volume resuscitation or restrictive resuscitation) and (hypovolemic shock or haemorrhagic shock or hemorrhagic shock or bleeding) and crystalloid fluid solution) or isotonic fluid solution or normal saline or NS) and Trauma*) or injur* or blunt trauma or penetrat* trauma or motor vehicle collisions or road traffic accidents or wound*). kf. not animal. af	56,102	201
Web-of-science	(Permissive hypotensive resuscitation) OR TOPIC: (hypotensive resuscitation) OR TOPIC: (controlled resuscitation) OR TOPIC: (balanced resuscitation) OR TOPIC: (low volume resuscitation) OR TOPIC: (restrictive resuscitation) AND TOPIC: (hypovolemic shock) OR TOPIC: (haemorrhagic shock) OR TOPIC: (bleeding) AND TOPIC: (crystalloid fluid) OR TOPIC: (isotonic fluid) OR TOPIC: (saline solution) OR TOPIC: (normal saline) OR TOPIC: (NS) AND TOPIC: (trauma) OR TOPIC: (injur*) OR TOPIC: (motor vehicle collisions) OR TOPIC: (road traffic accidents) OR TOPIC: (blunt trauma) OR TOPIC: (penetrat* trauma) OR TOPIC: (wound*) NOT TOPIC: (animal)	861,689	396
CENTRAL	(Permissive hypotensive resuscitation/OR hypotensive resuscitation/OR “controlled resuscitation”/OR balanced resuscitation/OR low volume resuscitation/OR restrictive resuscitation) AND (hypovolemic shock/OR haemorrhagic shock/OR bleeding) AND (Crystalloid fluid/ OR isotonic fluid OR Saline Solution/OR “Normal Saline”/OR “NS”) AND (Trauma*/OR injur*/OR blunt trauma/OR penetrat* trauma/OR motor vehicle collisions/OR road traffic accidents/OR wound*) NOT “animal”	42	42
PubMed	((Permissive hypotensive resuscitation[MeSH Terms]) OR hypotensive resuscitation[MeSH Terms]) OR controlled resuscitation[MeSH Terms]) OR balanced resuscitation[MeSH Terms]) OR low volume resuscitation[MeSH Terms]) OR restrictive resuscitation[MeSH Terms]) AND hypovolemic shock[MeSH Terms]) OR haemorrhagic shock[MeSH Terms]) OR hemorrhagic shock[MeSH Terms]) OR bleeding[MeSH Terms]) AND Crystalloid fluid/ solution[MeSH Terms]) OR isotonic fluid/ solution[MeSH Terms]) OR Normal Saline[MeSH Terms]) OR NS[MeSH Terms]) AND Trauma*[MeSH Terms]) OR injur*[MeSH Terms]) OR blunt trauma[MeSH Terms]) OR penetrat* trauma[MeSH Terms]) OR motor vehicle collisions[MeSH Terms]) OR road traffic accident[MeSH Terms]) OR wound*[MeSH Terms])) NOT animal[MeSH Terms]	389	125
Total		939,263	835

### Study selection

Titles and abstracts of the eligible elected studies were screened, and the eligible studies were assessed for accuracy, complete data entry, and the measurement of mortality rate among trauma patients. Studies that collected data from both normotensive and hypotensive trauma patients were included, as long as the data concerning the mortality rate of hypotensive patients could be extracted. The reason for including these studies was to comprehensively find all pertinent data from the existing clinical experimental studies. EndNote software version X7 (Thomson Reuters, 2013, USA) was used to eliminate duplicate studies.

### Data extraction

The data were extricated from the included articles by the main author of this review performed using a data extraction sheet, adapted from the handbook of Cochrane Library [18], for the following items: study details (author name, publication year, title of study, country of origin, and publication source); study characteristics (study design and aim); characteristics of participants and initial recorded variables [age, number of patients, baseline systolic blood pressure (SBP) and injury severity score (ISS); intervention and setting; and finally the outcome data (number of deaths among participants in the intervention and control groups)].

### Studied outcomes

The outcomes in this review were separated into two categories to obtain comprehensive measurement of the survival rate associated with the main intervention and the comparator. Thus; the primary outcome was in-hospital mortality during admission for the comparative studies that assess the survival rate between aggressive and restricted fluid resuscitation. The secondary outcome was the mortality rate of hypotensive fluid resuscitation of non-comparative cohort studies. The narrative form is provided for these outcomes of the included studies.

### Synthesis of data and statistical analysis

Survival rates among the participants in the included studies were calculated and expressed as a percentage; this percentage was compared between the two groups of patients resuscitated with low or high-volume crystalloid fluid. Due to the heterogeneity of the included studies, studies were sub-grouped depending on their primary focus into studies comparing the survival rate between low and large volume resuscitation, and non-comparative cohort studies that focussed on the mortality rates of hypotensive resuscitation. The odds ratio and hazard ratio were extorted from the eligible studies and used for the analysis for each group of studies. Additionally, the data imported from these studies included the 95% confidence interval (CI) and *P* values, in which *P* value of < 0.05 was deemed to represent statistical significance of the results.

### Assessment of bias

The Cochrane Collaboration criteria tool was used to assess the risk of bias of the randomised control trials (RCT); this tool examines sequence allocation, allocation concealment, blinding, incomplete outcome data address, selective reporting, and other bias [19]. The comparative and non-comparative cohort studies were assessed for bias based on the Methodological Index for Non-Randomized Studies (MINORS) scale, which encompasses eight assessed areas for non-comparative studies and 12 criteria for evaluating the included comparative studies [20]. Selection of these two tools was based on the quality of both the validity assessed and the ability to objectively score the risk of bias [20–22].

### Critical appraisal of the selected articles

In this qualitative systematic review, the Critical Appraisal Skills Program (CASP) tool was used to critically appraise the selected articles. Two types of CASP tools were followed; one tool of CASP for RCTs and another CASP tool for cohort studies [23]. This tool was selected for the following reasons; it is relevant to healthcare issues and clinical researchers, it is a simple-to-use tool for many reviewers, it enables the reader to objectively measure and examine each research element, it covers the most important components of scientific research, and it can be used for various research designs [24, 25].

## Results

### Search results

**Fig. 1:**
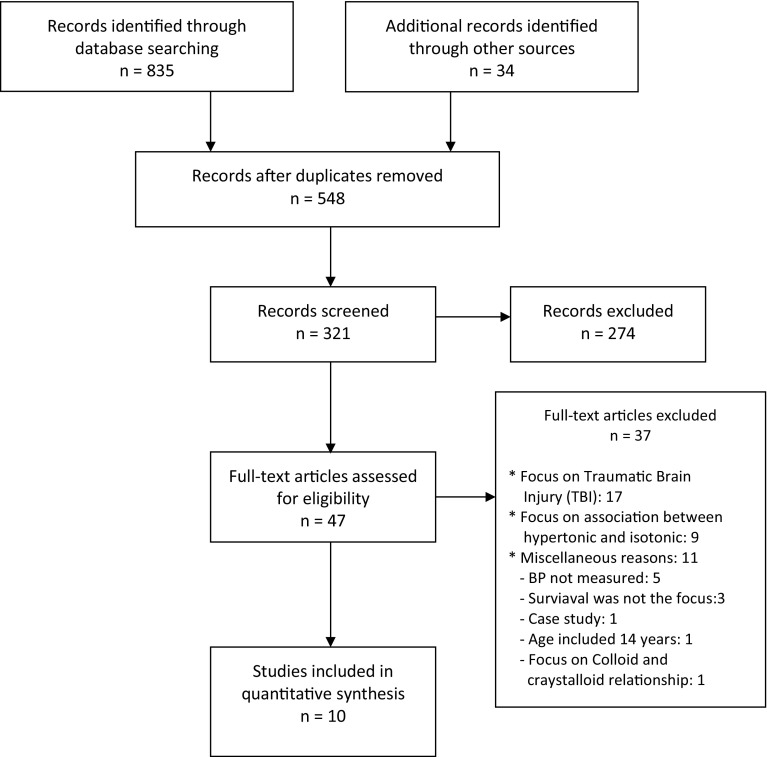
The applied PRISMA tool for the review

There were 869 articles initially identified as eligible studies for inclusion in the search process, as outlined in preferred reporting items for systematic reviews and meta-analyses (PRISMA) flowchart (see Fig. 1) [17]. 548 published articles were duplicates and eliminated from the list of included studies and a further 274 articles were exempt as they were irrelevant to this review due to a number of factors, including studies that were carried out on animals or, non-hypotensive patients, as well as those articles concerned with bleeding patients in non-emergency situations, such as gastric ulcers and chronic epistaxis (nasal bleeding). A total of 47 full-text studies were assessed for inclusion and it was found that 37 articles did not meet the eligibility criteria due to a number of factors including: case studies; studied that were carried out on patients with TBI, studies which focussed on the association between hypertonic fluid and isotonic fluid; studies that did not measure the survival rate or defined the initial records of SBP of the included participants; and concentration on the correlation between colloid and crystalloid. Ultimately, ten articles were selected and used in this qualitative systematic review.

### Characteristics of the selected studies

The selected studies for this review were five RCTs [13, 26–29], two prospective cohort studies [30, 31] and three retrospective cohort studies [32–34]. Seven studies were from the USA, two studies from Sweden and Australia, and one study was carried out in two countries; the USA and Canada. The RCTs and a prospective study were classified as comparative studies [13, 26–29, 31], which primarily aimed to compare the mortality rate between low and large volume resuscitation, whilst one prospective and three retrospective studies were non-comparative [30, 32–34]. Interestingly, the selected articles were conducted in various practice settings, including pre-hospital and in-hospital settings, such as emergency departments and operating theatres. The characteristics of the selected studies are summarised in Table 2.

**Table 2:** Characteristics of the included studies and the enrolled patients

Name, year	Hypotensive subjects (*N*)	Age range (mean)	Site	Location	Study type	Blood pressure and injury score	
						SBP (mmHg)	ISS
Bickell et al. [13]	598	20–40 (31)* years	Emergency department	Houston, USA	Randomised control trial	75.5**	26*
Dutton et al. [26]	110	17–42 (31)* years	Emergency department	Maryland, USA	Randomised control trial	107**	21.73**
Talving et al. [32]	102	22–55 (35.5)** years	Pre-hospital	Stockholm, Sweden	Retrospective (non-comparative)	U/S	28.5**
Morrison et al. [27]	90	15–45 (32.3)** years	Operating room	Houston, USA	Randomised control trial	75.85**	19.1**
Ley et al. [33]	Overall: 3137 H: 106	20–69 (37)* years	Emergency department	California, USA	Retrospective (non-comparative)	Overall: 133*	Overall: 10.3*
Kasotakis et al. [30]	1754	16–90 (43.5)* years	In-hospital	Multi-trauma centres, USA	Prospective (non-comparative)	111.1*	32.2*
Brown et al. [31]	Overall: 1216 H: 616	19–89 (40)* years	Pre-hospital	Multi-trauma centres: USA	Prospective (comparative)	68**	41*
Schreiber et al. [28]	192	≥ 15 years (41)* years	Pre-hospital	Multi-trauma centres: United States and Canada	Randomised control trial	82.7**	32.5**
Geeraedts et al. [34]	941	≥ 16 years	Pre-hospital	Sydney, Australia	Retrospective (non-comparative)	81*	13**
Carrick et al. [29]	168	15–45 (30)* years	Operating room	Houston, USA	Randomised control trial	82**	17.5**

*SBP* systolic blood pressure, *ISS* injury severity score, *U*/*S* unspecified, *mean, **median

### Level of evidence

The grading of recommendations, assessment, development, and evaluation (GRADE) framework, contains five levels of evidence, to describe the quality of evidence [35]. The enrolled studies ranged from randomised control trial (level 1), to retrospective studies (level 3).

### Ethical dimension of the selected studies

The selected articles were properly conducted after ethical approval, and ethical principles were considered during the study. The RCTs and prospective cohort studies included in this review excluded the informed consent of the participants due to either the clinical status of emergency patients or difficulties in explaining to the critically ill patients about emergency intervention, according to the federal regulations of the United States for Emergency Research [36, 37]. Two trials provided information about the study to the surrounding community and considered the community’s right to be withheld from the trial, as evident by issuing opt-out bracelets to the participants, who did not wish to be involved [26, 29].

### Description of the enrolled patients in the studies

A total of 4677 hypotensive patients were enrolled in the included studies, which recruited patients with either blunt or penetrating trauma. The included studies were conducted on adult patients aged from 15 to 55 years, except three studies that examined the hypotensive resuscitation on a mixed population of elderly and non-elderly trauma patients [30, 31, 33]. Two cohort studies included a mixed population of hypotensive and non-hypotensive trauma patients [31, 33] with a sum of 3617 non-hypotensive patients who were excluded from this review’s analysis. Additionally, 14 hypotensive elderly patients enrolled in one cohort study were directly excluded from analysis due to an uncalculated probability of death among hypotensive elderly participants [33]. Regarding the pre-registered record of SBP and ISS, one study computed the mean value of these variables for the whole cohort’s participants without specifying the mean SBP and ISS of the hypotensive group, which affected the reported result of the data [33]. Table 2 presents the summary of the characteristics of the enrolled studies.

### Assessing risk of bias of the included studies

By implementing the Cochrane assessment tool to assess the risk of bias of the RCTs, the quality of methodologies conducted was moderate. Due to the inability to obtain the statistical power of the results, one study was terminated, because of a statistically low sample size [29]. Through poor interventional blinding of the surgeons and anaesthesiologists in three RCTs [26, 28, 29], there was a potential source of ascertainment bias, which might cause error in the internal validity of the resultant findings [38]. Table 3 provides a summary of the findings yielded upon applying the criteria of Cochrane Collaboration to assess the risk of bias of the RCTs.

**Table 3:** Risk of bias assessment for randomized controlled trials (adapting cochrane collaboration criteria), and cohort studies (adapting MINORS scale)

Author, year	Cochrane collaboration tool					
	Adequate sequence generation	Allocation concealment	Blinding	Incomplete outcome data	Selective reporting	Other source of bias
Risk of bias assessment for cohort studies (adapting MINORS scale)						
Bickell et al. [13]	Probably	Probably	Unlikely	No	Unlikely	No
Dutton et al. [26]	No	Unlikely	Unlikely	No	Probably	No
Morrison et al. [27]	Unlikely	Probably	No	No	Probably	No
Schreiber et al. [28]	No	No	Unlikely	Yes	No	Probably
Carrick et al. [29]	No	No	Unlikely	Unlikely	Probably	Probably

Author, Year	A Clearly Stated aim	Inclusion of consecutive patients	Prospective collection of data	Endpoints appropriate to the aim of the study	Unbiased assessment of the study endpoint	Follow-up period appropriate to the aim of the study	Loss to follow up less than 5%	Prospective calculation of the study size	An adequate control group	Contemporary groups	Baseline equivalence of groups	Adequate statistical analyses	Total
Risk of bias assessment for cohort studies (adapting MINORS scale)													
MINORS items													
Talving et al. [32]	2	2	0	1	1	1	2	1	N/A	N/A	N/A	N/A	10/16
Ley et al. [33]	2	0	0	1	1	1	1	1	N/A	N/A	N/A	N/A	7/16
Kasotakis et al. [30]	2	0	2	2	1	2	2	1	N/A	N/A	N/A	N/A	12/16
Geeraedts et al. [34]	2	0	0	2	1	1	2	1	N/A	N/A	N/A	N/A	9/12
Comparative cohort study													
Brown et al. [31]	1	0	2	2	1	2	0	1	1	2	1	1	14/24

*N/A* not applicable

*The items are scored as following: 0 = not reported, 1 = reported but not adequate, 2 = reported and adequate

On the other hand, using the MINORS scale gave results that ranged from seven to 12 out of 16 for the non-comparative cohort studies, and the counted score was 14 out of 16 for the prospective comparative study, as illustrated in Table 3. These results showed a moderate risk of bias among the selected non-randomised studies. Notably, all of the included cohort studies enrolled adequate representative sample sizes of post-trauma patients, which is attributed to the nature of data collection by retrieving the information from patients’ records and the ease of obtaining ethical approval from review boards. Applying the tool showed that one retrospective study revealed a high risk of bias due to the inappropriate use of analysis tools whilst measuring the elicited data, and misclassification between elderly and non-elderly patients [33]. In addition, one comparative study failed to obtain adequate equivalence between the enrolled patients in the low volume group (with 123 enrolled patients) and the control group (480 patients) [31].

### Critiquing the studies

Upon applying the CASP tool to this review, the critical appraisal of the comparative studies showed that Bickell and his colleagues conducted a trial with poor randomisation methodology with a potential source of selective bias of some participants [13], in addition to a high rate of data loss during follow-up in another RCT [29], which might affect the merit of statistical analysis in the current review. The sample size of the RCTs was relatively small in contrast to the large sample size recruitment in the cohort studies, which might be reasonable due to several factors concerning issues related to ethical and economic aspect [39]. Notably, all of the included non-comparative cohort studies lacked rigorous statistical analysis and adjusting of the confounders, which might breach the internal validity of these studies and influence the reported findings [40].

## Mortality rate of hypotensive versus aggressive fluid resuscitation in the comparative studies

The included RCTs showed that the mortality rate in 1157 trauma patients resuscitated with low and large volumes of fluid were 21.5% (123 deaths from 570 patients) and 28.6% (168 deaths from 587 patients), respectively (Table 4). Interestingly, the results of only one trial reported a statistical significance of a high mortality rate associated with immediate aggressive fluid resuscitation of hypotensive traumatised patients (*P* = 0.04) [13]. However, the prospective comparative study revealed an increased proportion of death in the hypotensive group to 15.4% compared to a 3.75% death rate in the aggressive resuscitation group, which might result from the unequal sample size between the two comparator groups [31]. Collectively, the results of six comparative studies reported that the survival rate is slightly higher in the conservative fluid resuscitation group with a mean value of 82.9%, whilst the survival rate amounted to 80.2% in post-trauma patients resuscitated with large volume of fluids (see Table 4).

**Table 4:** Pooled mortality rate between hypotensive and aggressive resuscitation groups in the comparative studies

Name, year	Sample size of hypotensive patients	No of deaths		*P* value	Hazard ratio	Survival rate between restrictive versus large volume resuscitation (%)
		Death/patients (hypotensive group) (%)	Death/patients (aggressive group) (%)			
Randomised control trials and prospective study						
Bickell et al. [13]	598	86/289 (29.7)	116/309 (37.5)	0.04	N/M	70 versus 62
Dutton et al. [26]	110	4/55 (7.2)	4/55 (7.2)	N/M	HR 1.00	92.7 versus 92.7
Morrison et al. [27]	90	10/44 (22.7)	13/46 (28.2)	0.58	HR 1.10	77.2 versus 71.7
Schreiber et al. [28]	191	5/96 (5.2)	14/95 (14.7)	N/M	aOR 0.39	94.8 versus 85.2
Carrick et al. [29]	168	18/86 (20.9)	21/82 (25.6)	0.47	HR 0.48	78.5 versus 73.7
Total	1157	123/570 (21.57%)	168/587 (28.6%)			
Prospective cohort study						
Brown et al. [31]	603	19/123 (15.4%)	18/480 (3.75)	0.90	HR 0.81	84.55 versus 96.25

Groups	Population (*n*)	Mortality *n* (%)	Survival rate (mean)
Calculation of overall mortality rate and survival rate			
Hypotensive resuscitation	693	142 (20.49)	82.95%
Aggressive resuscitation	1067	186 (17.43)	80.25%

*aOR* adjusted odds ratio, *n* number, *vs*. versus

## Mortality rate of hypotensive fluid resuscitation in the non-comparative studies

To attain findings that are more meaningful from this study and due to presumed heterogeneity, which resulted from the calculation method of mortality rate and an unspecified amount of fluid administration in the hypotensive group, subgroup analysis of these studies was implemented. The result of one retrospective study was impressive, and concluded that crystalloid fluid resuscitation using more than 1.5 l was highly associated with the mortality rate of non-elderly trauma patients [Odds ratio (OR) 2.09, 95% CI 1.31–3.33, *P* = 0.002] (Table 5) [33]. The remaining non-comparative studies reported that the overall mortality rate was 279 deaths from 2797 patients, which amounts to 9.97% (Table 5) [30, 32, 34]. Notably, one prospective study was performed on both low volume and large volume (1 ml–15 l), without specifying the volumes administered and resultant risk of death in each group of fluid volumes, which make the results of this study inconclusive and indecisive [30].

**Table 5:** Mortality rate of fluid resuscitation strategies in the non-comparative studies

Name, year	Sample size of hypotensive patients	No of patients given less amount of fluid	Amount of administered fluid	Overall no of death among hypotensive patients	Odds ratio	*P* value	95% CI
Uncalculated number of deaths							
Ley et al. [33]	106	N/S	1 ml–1.5 l	N/S	OR 2.09	0.002	1.31–3.33
Calculated deaths number							
Talving et al. [32]	102	16	(1 ml–500 ml)	31 (30%)	OR 0.7	N/M	0.07–5.9
Kasotakis et al. [30]	1754	N/S	(1 ml–15 l)	159 (9.1%)	N/M	0.275	N/M
Geeraedts et al. [34]	941	253	(500 ml–1 l)	89 (9.5%)	OR 0.97	0.93	0.45–2.09
Total	2797			279 (9.97%)			

*N/S* not specified, *N/M* not measured, *OR* Odds ratio

## Discussion

Despite significant advancement in therapeutic emergency management of trauma patients, there is a dearth of experiments that clinically examine the impacts of permissive low-volume resuscitation on hypotensive patients following trauma. This review was to critically evaluate the available literature that assessed the survival rates associated with deliberate hypotensive resuscitation in traumatised patients with low blood pressure. This paper has evaluated ten articles as a result of a systematic comprehensive search of all published literature on the hypotensive resuscitation topic.

The results from high-quality evidence have shown that the mortality rate of comparative randomised trials was lower among patients resuscitated with low-volume fluids (21.5%), compared with large-volume resuscitation (28.6%). This finding was impressive and meaningful but the result was elected from trials that laced strong methodology, in which one study was carried out with improper allocation concealment [13]; furthermore, one trial was performed with insignificant statistical power, thus it was terminated based on the decision of the Data Safety Monitoring Board [29].

Bearing in mind, the collective comparative studies that encompassed five RCTs and one prospective study showed that the pooled survival rate of limited versus liberal volume resuscitation was 82.9 and 80.2%, respectively. This finding was statistically insignificant, which could be attributed to the poor sampling distribution of the prospective sample, as evidenced by unequal sample size between the two groups with only 20% enrolled patients in the low-volume resuscitation. This could have considerable influence on the accuracy of the final results [41]. In addition, the enrolled comparative prospective study was conducted on near-death patients; as evident by recruiting patients having high ISS, with a mean value of 41 [31].

Moreover, the analyses of non-comparative studies were divided into two groups due to the existing heterogeneity between cohort studies in terms of the statistical measurement of death rates and in defining the amount of administered fluid. One retrospective study concluded that patients treated with low volumes of fluid (less than 1.5 l) were less likely to die [35]. This remarkable finding was similar in the remaining three non-comparative studies that exposed the pooled mortality rate of the entire cohort was 9.97%. However, this result requires further interpretation and profound exploration due to the vagueness of which fluid resuscitative strategies were examined, which might not be a conclusive result.

Additionally, the results, from the drawn samples of traumatised adults aged from 15 to 55 years, have shown that this younger adult group have better survival when resuscitated with low fluid compared geriatric patients. In one retrospective study, elderly trauma patients (< 70 years), who were managed with large-volume (more than 1.5 l) have shown worsen survival rate compared with younger adults [33]. However, this end requires more randomised trials to investigate the reliability of such result on both children and elderly patients.

To summarise, the inference that can been drawn from the preliminary analysis of the comparative and non-comparative studies is that there is a non-significant trend toward improved survival rate after administering hypotensive fluid resuscitation in hypotensive trauma patients. In addition, the pooled mortality rate of the selected studies shows an increased mortality rate among patients resuscitated with large-volume resuscitation, thus this strategy can worsen the resulting health outcomes of trauma patients. This finding can be attributed to the increased coagulopathy status and massive bleeding rate following aggressive resuscitation due to interim raised blood pressure and heart rate readings, which hinder the early recognition of shock signs [12, 42–44]. However, this result is contraindicated by some authors who claimed no beneficial effects on the survival rate via fluid administration, as it might delay the definitive surgical control of bleeding and impair blood flow to the vital organs [45, 46].

This review’s finding is consistent with the studies that tested the same outcome of death rate on animal models, which revealed decreased mortality rates of animals treated with hypotensive resuscitation [16, 47–50]. Not surprisingly, evidence from lab studies emphasised that there are several complications associated with large volumes such as tissue oedema, diminished inflammatory responses, metabolic acidosis and hypoxemia, and cardiac and respiratory impairment [12, 51].

## Limitations

This review presumably has inherent bias in the list of eligible studies, but this limitation was avoided by a systematic search of the potential articles of interest. In addition, the selected studies were heterogeneous, in which they differ from each other in the studied population, nature of the targeted outcomes and analysis tools used in each study. The included studies ranged from moderate to high quality study designs according to the GRADE methodology [35]. This affected the consistency of the results drawn from various methodological designs and the strength of the drawn conclusion.

Additionally, this paper is limited by including studies that lack pre-defined clinical parameters and markers of hypovolemic shock other than blood pressure values. Defining hypovolemic shock based on the blood pressure value is very liberal and unsuitable, which may misdirect the treating trauma physicians and lead to further compromise of the patient’s condition [9, 51]. The labelling of patients with low blood pressure with a diagnosis of haemorrhagic shock, may diminish the quality of care delivery; thus fluid therapy should be tailored for each individual based on their current state of shock. Critical clinical judgment of diagnosing a patient with profound shock should rely on assessing various vital signs and the patient’s clinical condition, as advised by the advanced trauma life support (ATLS) system, which proposed a unique grading scoring system that aids the classification and prompt recognition of the state of hypovolemic shock [52].

Some of the selected articles in this paper measured 24-h mortality rate and others focussed on calculating the death rate during the entire period of hospital admission, which created further confusion during analysis. Elimination of this shortcoming was attempted before commencing the search method; unfortunately, this was unresolved due to the paucity of existing empirical evidence on this topic and the disparity between the studies included in this review concerning their measurements of the mortality rate as being either within 24 h or during hospital admission of trauma patients.

Nonetheless, the enrolled studies tested fluid therapy on trauma patients without defining the end point’s exact amount of crystalloid fluid. Titrating the administration of crystalloid fluid to maintain a low-pressure value in trauma patients was considered the landmark of the targeted volumes to maintain hypotension during trauma resuscitation in the previous empirical studies [53, 54]. Several studies recommended giving on-demand boluses of small volumes of fluid ranging from 250 to 500 ml, which is sufficient to sustain hypotension before definitive control of bleeding by surgical intervention [55, 56].

## Future implications

The importance of drawing conclusions from critiquing and meta-analysis of the literature is to add to the body of knowledge in trauma science and use that knowledge to inform healthcare decision-makers. The current knowledge is unable to ascertain whether hypotensive resuscitation is safe or not, which entails further studies that will help to guide current trauma practice concerning crystalloid fluid administration. Future trauma research should concentrate on conducting more trials on post-traumatic patients in pre-hospital, as well as in-hospital settings to obtain absolute answers on the efficacy of PHR. In addition, investigation into early versus late death after PHR would be advisable in future studies, to have better awareness of the potential risks of early or late death. The conduct of future trials should be conducted with large sample sizes, proper randomisation methodology, and more precise amounts of fluid that define the limited-volume strategy.

## Conclusions

In conclusion, this paper has included ten studies that investigated fluid resuscitation of hypotensive patients, who sustained trauma. The results of these studies demonstrated that the associated mortality rate induced by hypotensive resuscitation is low, compared with aggressive resuscitation. Critical appraisal of the included studies revealed that comparative and non-comparative studies were carried out with moderate to strong quality, but with evidence of poor methodology and concealment procedure. This finding could confirm that administrating large volumes of crystalloid fluid should be cautiously practiced in the clinical setting. In summary, the current review is supportive of previous empirical studies in showing that managing traumatic haemorrhagic shock patients with the PHR strategy is a feasible and safe practice in pre-hospital and in hospital settings.
